# Comparing food limitation among three stages of nesting: supplementation experiments with the burrowing owl

**DOI:** 10.1002/ece3.616

**Published:** 2013-07-05

**Authors:** Troy I Wellicome, L Danielle Todd, Ray G Poulin, Geoffrey L Holroyd, Ryan J Fisher

**Affiliations:** 1 Department of Biological Sciences, University of AlbertaT6G 2E9, Edmonton, Canada; 2 Canadian Wildlife ServiceRoom 200, 4999–98th Ave., T6B 2X3, Edmonton, Canada; 3 Department of Biology, University of ReginaS4S 0A2, Regina, Canada; 4 Royal Saskatchewan Museum2340 Albert St., S4P 2V7, Regina, Canada

**Keywords:** Brood reduction, fledgling production, fledgling size, grassland, nestling survival, raptor, timing food limitation

## Abstract

Food availability is an important limiting factor for avian reproduction. In altricial birds, food limitation is assumed to be more severe during the nestling stage than during laying or incubation, but this has yet to be adequately tested. Using food-supplementation experiments over a 5-year period, we determined the degree and timing of food limitation for burrowing owls (*Athene cunicularia*) breeding in Canada. Burrowing owls are an endangered species and food limitation during the nestling stage could influence reproductive performance of this species at the northern extent of their range. Supplemented pairs fledged on average 47% more owlets than unfed pairs, except during a year when natural food was not limiting (i.e., a prey irruption year). The difference in fledgling production resulted from high nestling mortality in unfed broods, with 96% of all nestling deaths being attributed to food shortage. Supplemental feeding during the nestling period also increased fledgling structural size. Pairs fed from the start of laying produced the same number of hatchlings as pairs that received no supplemental food before hatch. Furthermore, pairs supplemented from egg laying to fledging and pairs supplemented during the nestling period alone had the same patterns of nestling survival, equal numbers of fledglings, and similar fledgling mass and structural size. Our results provide empirical support for the hypothesis that the nestling period is the most food-limited phase of the breeding cycle. The experimental design we introduce here could be used with other altricial species to examine how the timing of food limitation differs among birds with a variety of life-history strategies. For burrowing owls, and other species with similar life histories, long-term, large-scale, and appropriately timed habitat management increasing prey abundance or availability is critical for conservation.

Our results provide empirical support for the hypothesis that the nestling period is the most food-limited phase of the breeding cycle. For burrowing owls, and other species with similar life histories, long-term, large-scale, and appropriately timed habitat management increasing prey abundance or availability is critical for conservation.

## Introduction

Food supply is widely regarded as one of the most important factors determining the amount of investment in breeding by birds (Martin 36; Robb et al. 53), and within a breeding season, reproductive output could be food limited during egg formation, incubation, and/or brood rearing (Martin 36). For altricial species, parental feeding of young often imposes the most severe energetic bottleneck (Murphy and Haukioja 39; Bryant and Tatner 6; Brinkhof and Cavé 5). Accordingly, Lack (31) advocated that laying date, clutch size, hatching asynchrony, and brood reduction were all adapted to food limitation during the nestling period. In contrast, other empirical work with altricial species has shown reproductive limitation early in the breeding season, via proximate energetic constraints on egg-laying females (Perrins 44; Korpimaki 28; Nilsson 40). Further still, some indirect evidence suggests that incubation is the most food-limited stage for certain species (e.g., Mertens 37; Siikamaki 55; Gende and Willson 17; Reid et al. 50). In recent decades, increased human modification in habitat (Brennan and Kuvlesky 4) and changes in seasonal conditions as a result of climate change may be exacerbating the degree of food limitation, or modifying its timing, for many birds (Evans 15; Both et al. 2).

Because manipulation of dietary intake is the most direct way to address questions of food limitation (Martin 36), supplemental feeding has been used frequently in avian breeding experiments (Boutin 3). However, given that the nestling period is often assumed to be the most energy-limiting stage of breeding for most altricial species, surprisingly few experiments have provided extra food solely during that stage. Rather, in most investigations, food supplementation starts before egg laying and continues through the reproductive cycle (e.g., Hogstedt 23; Davies and Lundberg 11; Arcese and Smith 1; Harrison et al. 20). Consequently, effects of food limitation during the nestling phase cannot be separated from effects during other time periods (Dunn et al. 13). Only a handful of studies in altricial species provided extra food to broods during the nestling period alone (Simons and Martin 57; Richner 51; Garcia et al. 16; Verhulst 62; Gende and Willson 17; Wiehn and Korpimaki 67; González et al. 19). These food-supplementation studies ranged from 1 to 14 years in duration, and most found some evidence of food limitation. Unfortunately, demonstrating that food intake during the nestling period affects the number and/or size of fledglings gives no indication of the importance of food availability in the nestling stage relative to availability in other stages of the nesting cycle (Wiehn and Korpimaki 67). To accomplish this, supplementation must begin at different stages in the breeding cycle.

Here, we present results from food-supplementation experiments in an intensely human-modified agricultural setting conducted over 5 years with the burrowing owl *Athene cunicularia*, Molina. To test if food typically limits reproduction during the nestling period, we compared the quality (size and mass) and quantity of fledglings between control (unsupplemented) pairs and pairs provided with extra food from hatching until fledging. In two of the years, we also fed a third subset of owl pairs from clutch initiation until fledging. To our knowledge, no previous experiment has compared fledgling production by individuals that were food supplemented during the nestling stage with those that were supplemented through the egg laying, incubation, and nestling stages.

The burrowing owl is an endangered species in Canada (COSEWIC 9), a species of conservation concern in the United States (Klute et al. 27), and a species of special protection in Mexico (Klute et al. 27; SEMARNAT 54). Although burrowing owls nest and forage in a variety of habitat types (e.g., native and tame pastures, golf courses, roadside ditches, mowed lawns, and airport fields; Poulin et al. 48; Marsh 34), reduced prey availability or accessibility during the nesting season could be one of the reasons for recent population declines at the northern extent of their range (Poulin et al. 47; COSEWIC 9).

Ultimately, knowledge of if and when food may limit reproductive output in avian populations is essential for determining proper timing and management of habitat, or in an extreme case, when to supplement food for wild populations. In addition, understanding when food typically limits reproductive output during the breeding cycle has important implications given the current focus on climate change–induced mismatches between food availability and breeding phenology (Both et al. 2; Dunn et al. 13).

## Materials and Methods

### Study area and study species

As part of a long-term (1993–2010) burrowing owl monitoring study in the grassland ecoregion of Canada, we conducted our supplementation experiments on burrowing owls in 1992, 1993, and 1996–1998 on a 10,000 km^2^ study site (49°40′–50°35′N, 103°45′–105°40′E). The owls in our study area nested in heavily grazed pastures that were interspersed among other types of agricultural fields (e.g., cropland and hay fields). Normally, owls nest in underground chambers at the ends of tunnels excavated by Richardson's ground squirrels *Urocitellus richardsonii* Sabine or American badgers *Taxidea taxus* Schreber. In our study, the majority of pairs nested in artificial burrows (see Johnson et al. 26; Figure 5 for a detailed description of artificial burrow design). Artificial burrows were opened during each nest visit to determine clutch initiation date (CID), hatching date for each egg, and the number of eggs or owlets. From 15 days before egg laying to 35 days after hatching, we also counted vertebrate prey stored in nest chambers (see Wellicome 64). Much of this information could not be collected for pairs in natural burrows because their nest chambers were inaccessible. We were, however, able to determine the number of young produced at each natural nest by counting the maximum number of owlets observed during three or more observation periods late in the nestling stage. Nestling mortality was quantified only for broods nesting in artificial burrows.

#### Nesting chronology

In our study population, clutches are initiated in the first half of May, with egg laying lasting 8–17 days depending on clutch size. First hatch occurs 15–22 days after clutch completion and hatching of all eggs continues over a 1- to 7-day period. Nestlings are brooded for the first 2 weeks post hatch, after which they become more mobile. We considered each nestling to have fledged when it reached 41 days old (Wellicome 63). Age of young was estimated at natural burrows based on feather development (Priest 49) or by capturing and measuring owlets and comparing measurements to those of known-age owlets from artificial burrows (see Material and Methods section). The calculated ages of the oldest nestlings in natural burrows provided estimates of hatching date for each brood. We were able to unambiguously determine nesting chronology in artificial burrows.

#### Nestling measures

Nestlings in artificial burrows were marked with a unique combination of colors by applying indelible ink to feathers on the insides of their legs. Color combinations were maintained until nestlings were 16 days old, after which nestlings were fitted with numbered U.S. Fish and Wildlife Service aluminum bands. Each nestling was weighed, and tarsus, culmen, and wing chord (mm) lengths were recorded every second day during hatching, and every third (1992) or sixth day (1993 and 1996) thereafter, until they were 41 days old. Male and female burrowing owls are externally indistinguishable so we could not examine sex differences in nestling measurements. Because we had difficulty recapturing birds when they were exactly 41 days old, we fitted each individual's morphometric measurements over time to separate logistic growth curves and used the resulting equation to interpolate measures for the individual at age 41.

Lengths of tarsus, culmen, and wing for each individual at age 41 were incorporated into a principal components analysis (PCA). Each bird's score on the first component (hereafter “PC1”) of the PCA served as a measure of its structural size at fledging.

### Feeding experiments

#### Egg laying until fledging

To control for effects of CID on egg production, pairs were alternately assigned to supplemented and control groups according to their CID. Every third pair in 1993, and every second pair in 1996, nesting in an artificial burrow was assigned to be food supplemented; all other pairs nesting in artificial burrows were not supplemented. In 1996, additional pairs nesting in natural burrows, where CID could not be determined, were included in the experiment. These pairs were randomly assigned to either the supplemented or control group according to their spring-arrival date, which is highly correlated with CID (Wellicome 63). Supplemental feeding of pairs nesting in artificial burrows began after the first egg (24 pairs) or the second egg (8 pairs) had been laid, whereas feeding started in the second week of May for pairs in natural burrows (2 pairs, 1996). Fed pairs were provided with 255 g of white laboratory mice every third day (a rate of 85 g/pair/day), which is >3 times the metabolic requirements for daily existence of an adult burrowing owl in captivity (mean = 26 g/day; Marti 35). To ensure that only intended recipients had access to supplemented food, food was placed in the tunnel of each nest ≥60 cm from the burrow entrance. Supplemental feeding continued until all nestlings fledged or until the nesting attempt failed. Owls were observed eating supplemented food immediately after it was provided, remains of laboratory mice were found inside nest chambers, pure white fur was found in regurgitated pellets at all supplemented nests, and supplemented pairs had larger prey caches and regurgitated more pellets compared with control pairs (Wellicome 63), suggesting that owls readily accepted supplemented food. Control pairs were also visited every third day and disturbed for the same duration as supplemented pairs.

#### Hatching until fledging

Half of all pairs nesting in artificial burrows that were unfed during the prehatch periods were fed during the nestling period. To ensure that pairs in this treatment group had the same average number of hatchlings as those pairs in control groups, nests were ranked by clutch size and hatching date, then alternately assigned to each treatment. Supplemental feeding of pairs in natural burrows began in the first week of June in 1992, 1997, and 1998, and in the second week of June in 1996 because hatch was later that year (see Material and Methods). Supplementation of pairs in artificial burrows commenced immediately after hatch. All supplemented pairs were provided with 255 g of food at 3-day intervals for the duration of the nestling period. In the first half of the nestling period, laboratory mice were used for supplemental feeding, but in the second half, a combination of laboratory mice and juvenile quail was used. Food remains (tails, feathers, and bones) in regurgitated pellets of fed pairs confirmed that the owls were eating both quail and laboratory mice.

### Return rates

Each year we captured breeding owls within the study area. A small proportion of these recaptured individuals had been banded as nestlings. We calculated a basic return rate of nestlings from pairs that were either fed or unfed. We suspect that many of the owlets produced on our study area permanently disperse and breed elsewhere (Duxbury 14; Macias-Duarte 33); however, our index of recruitment should indicate whether individuals from supplemented nests returned at least as often as individuals from unfed pairs.

### Data analyses

#### Prey cache

We only included information on prey cache size from unfed pairs in artificial burrows. We calculated the mean vertebrate cache size per nest visit, and then calculated an overall annual mean. Annual variation in vertebrate prey cache size was examined, after log-transforming prey counts, using an ANOVA (year as a fixed factor). If year had a significant effect on prey cache size, we used Tukey's tests to compare differences between years (α = 0.05). All statistical tests were performed using Systat v. 11.0 (Systat Software, Inc., Chicago, IL).

#### Feeding experiments

As this study is concerned with the effects of food limitation on reproductive output and nest failures were random with respect to feeding treatments (Wellicome 63), we excluded nests that completely failed. Of the 224 adult owls in this study, 61% were color banded and none of the banded birds returned to breed more than once during our study. Therefore, we assumed that unbanded birds were also included once in the study and that all breeding events were independent.

Proportion of hatchlings that fledged was arcsine transformed, otherwise all other response variables were not transformed. ANOVAs were used to test whether variation in the number of hatchlings or the mass and size of owlets were influenced by: treatment (unfed and fed from hatching to fledging), year, and their interaction. When examining differences in response variables between control pairs and pairs supplemented for the nestling period, data from all 5 years were included, except where fledgling mass and size were involved as these were only collected in 1992, 1993, and 1996. Because we also collected data on hatching dates and number of fledglings from both natural and artificial burrows, we included “type of nest burrow” as an explanatory variable.

Because supplemental feeding from egg laying through fledging only occurred in 1993 and 1996, we used those 2 years to test for reproductive differences among the three treatment groups, year, and a treatment *x* year interaction. Interaction terms were initially included in ANOVA models, but were subsequently excluded if nonsignificant (*P *> 0.05). In such cases, *F*- and *P*-values from the model without the interaction are presented. Test statistics for all ANOVAs were based on Type III sums-of-squares. When significant effects were detected, we performed pairwise multiple comparisons with Tukey's tests. Because sample sizes were small, we increased statistical power by setting ∝ = 0.10 when testing fledgling measures; significance was determined if *P *≤ 0.05 in all other analyses. We calculated power (using observed parameter values) whenever *P* was ≥0.05 but ≤0.20.

#### Return rates

There were few returns of individuals banded as nestlings, therefore we only present descriptive information on return rates of nestlings in fed and unfed treatments and pool the two feeding treatments.

## Results

### Prey cache

**Figure 1:**
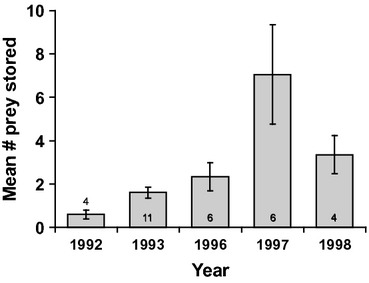
Annual variation, from May to July, in vertebrate prey cache size for burrowing owls nesting in artificial burrows that did not received supplemental food (mean ± SE). Values at the base of each bar indicate total number of nests from which prey cache size information was collected.

Mean number of vertebrates cached at control nests varied significantly among years (*F* = 9.192, *P *< 0.001; Fig. 1). Cache sizes were between 3 and 12 times larger in 1997 compared to 1992, 1993, and 1996 (Tukey multiple comparisons, all *P *< 0.02; Fig. 1); mean prey cache size was approximately twice as large in 1997 compared to 1998, but this difference was not statistically significant (*P *= 0.31). Caches were larger in 1998 compared to caches in 1992 (*P *= 0.03), but not compared to caches in 1993 or 1996 (*P *> 0.30).

### Feeding experiments

#### Hatching

Hatching date did not differ between controls and pairs that were fed from hatching to fledging (*F *= 0.07, *P *= 0.79; Table 1), nor did hatching date differ according to burrow type (*F *= 1.04, *P *= 0.31); however, hatching date differed among years (*F *= 6.26, *P *< 0.001; Table 1). Mean hatching date was later in 1996 compared to all years (Tukey tests, *P *≤ 0.02) except 1997 (*P *= 0.29), and later in 1997 than 1998 (*P *= 0.05; Table 1). When hatching dates were compared among all three experimental groups in 1993 and 1996, there was no effect of treatment (*F *= 0.70, *P *= 0.50; Table 1), nor was there a year-by-treatment interaction (*F* = 0.50, *P* = 0.61), but there was a significant effect of year (*F* = 16.5, *P *< 0.001; Table 1).

**Table 1:** Mean ± SE (sample size) hatching dates (June 1 = 1) for broods of food-supplemented and control burrowing owl pairs over 5 years in Saskatchewan, Canada

Treatment	1992	1993	1996	1997	1998
Unfed controls	6.7 ± 2.8 (6)	8.0 ± 1.6 (11)	17.0 ± 3.7 (6)	10.3 ± 1.9 (10)	7.8 ± 1.5 (8)
Fed (hatching to fledging)	8.4 ± 1.0 (5)	9.3 ± 1.5 (10)	13.8 ± 2.9 (9)	11.8 ± 1.8 (12)	5.9 ± 1.1 (21)
Fed (laying to fledging)	–	10.3 ± 1.8 (14)	17.3 ± 1.5 (16)	–	–

Hatching date (i.e., date first nestling of brood hatched) was recorded for all 111 broods in artificial burrows, and was estimated for 17 additional pairs nesting in natural burrows (see Methods: Material and Methods). No broods in 1992 and 1997–1998 were fed from laying until fledging.

**Figure 2:**
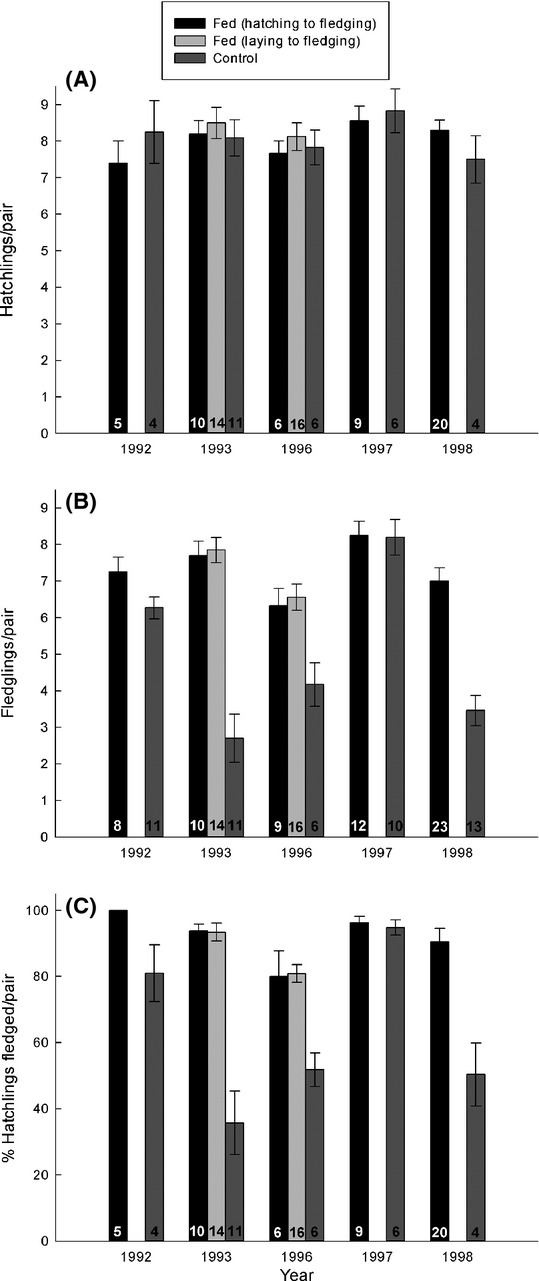
Mean (±SE) number of hatchlings (A), number of fledglings (B), and percentage of hatchlings fledged (C) per successful nest, for burrowing owl pairs in three experimental treatments. Pairs supplemented with food from hatching until fledging are shown in relation to control pairs in five study years. In 1993 and 1996, a third treatment of pairs supplemented with food from egg laying until fledging occurred. Sample sizes are presented at the base of each bar. Number of fledglings is given for pairs in both natural and artificial burrows, but number of hatchlings and percentage of hatchlings fledged could only be determined for pairs in artificial burrows.

Number of hatchlings did not differ among years (*F* = 1.11, *P* = 0.36), nor between controls and pairs fed during the nestling period (*F* = 0.06, *P* = 0.81), and there was no significant year-by-treatment interaction (*F* = 0.61, *P* = 0.66; Fig. 2A). When owls were also fed during the laying, incubation, and nestling stages (1993 and 1996), number of hatchlings did not differ among the three experimental groups (*F* = 0.49, *P* = 0.61) or between years (*F* = 1.01, *P* = 0.32), and there was no year-by-treatment interaction (*F* = 0.04, *P* = 0.97; Fig. 2A).

#### Quantity and quality of fledglings

Pairs that were fed from the time of hatching until fledging produced on average 2.4 more young compared with controls (*F* = 53.9, *P *< 0.001; Fig. 2B), with no effect of burrow type (*F* = 0.59, *P* = 0.44). Mean number of fledglings produced per nest significantly differed among years (*F* = 15.9, *P *< 0.001; Fig. 2B). In 1997, fledgling production was higher compared to the other 4 years (*P *≤ 0.02 for all pairwise comparisons); however, there was also a significant interaction between year and treatment (*F* = 8.5, *P *< 0.001; Fig. 2B). Feeding treatments in 1993, 1996, and 1998 all resulted in pairs fledging more offspring than unfed pairs (pairwise comparisons, *P *< 0.01). However, feeding treatments resulted in no difference in offspring production in 1992 or 1997 (pairwise comparisons, *P* > 0.70), although in 1992 fed pairs produced, on average, one more fledgling than unfed pairs (Fig. 2B).

In 1993 and 1996, when some pairs were also fed from egg laying through fledging, treatment (unfed, fed from hatch until fledging, and fed from laying until fledging) significantly influenced the number of fledglings per pair (*F* = 33.76, *P *< 0.001; Fig. 2B). Fledgling production for pairs fed through the egg laying, incubation, and nestling periods was no higher than for pairs fed during the nestling period alone (*P* = 1.00), but pairs fed from hatching to fledging and those fed from egg laying through fledging fledged 3.6 and 3.8 more young, respectively, than did unfed pairs (Tukey test, *P *< 0.001; Fig. 2B). There was no difference in fledgling output between the 2 years (*F* = 1.04, *P* = 0.31), but there was a significant year-by-treatment interaction (*F* = 4.73, *P* = 0.01). In 1993, pairs in both feeding treatments produced more fledglings than pairs not fed (Tukey pairwise comparisons, *P* < 0.01; Fig. 2B). Pairs that were fed from laying to fledging in 1996, produced more fledglings than pairs that were unfed (Tukey pairwise comparisons, *P *< 0.05). Although pairs that were fed from hatching to fledging produced 2.2 more fledglings than unfed pairs, this was not statistically significant (*P* = 0.098; Fig. 2B). Analyses examining variation in the percentage of hatchlings fledged (Fig. 2C) in response to treatment and burrow type mirrored those examining the number of fledglings produced and so are not presented.

**Figure 3:**
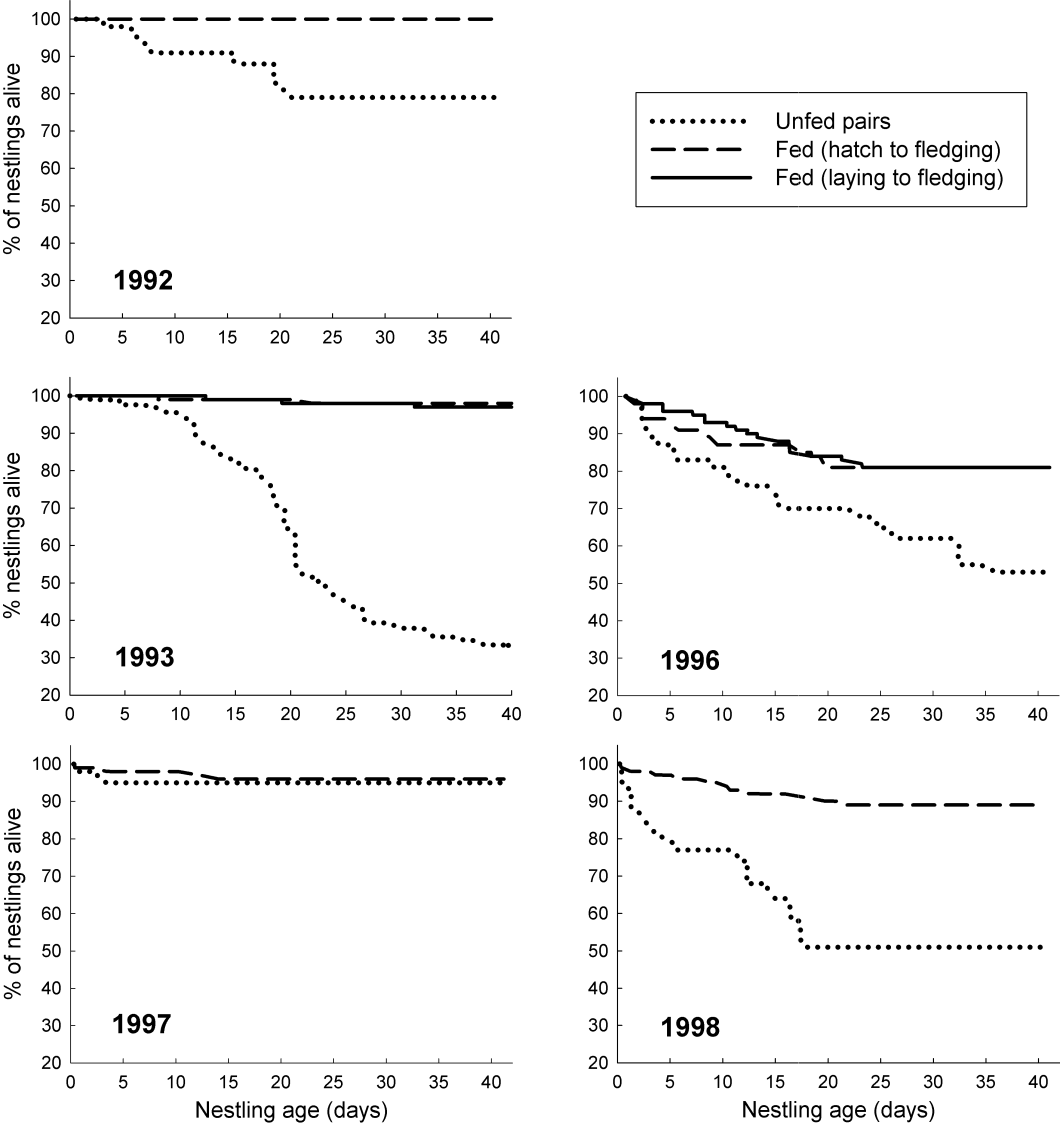
Survival of burrowing owl nestlings, from age 0 to 41 days (age 0 = individual's hatch day), in each experimental group (artificial burrows only). The numbers of hatchlings in “Unfed” and “Fed (hatching to fledging)” groups, respectively, were 33 and 37 (1992), 89 and 82 (1993), 47 and 46 (1996), 53 and 77 (1997), and 31 and 164 (1998). The number of hatchlings in the “Fed (laying to fledging)” treatment was 119 in 1993, and 130 in 1996.

Unfed owlets died in the first half of the nestling period in 1992, 1997, and 1998, but mortality was unrelated to age in 1993 and 1996 (Fig. 3). Of all deaths (*N* = 176), 169 (96%) were attributed to food shortage. Ninety-six of these 169 nestlings had been weighed within 5 days of their death. Fifteen percent of these showed normal patterns of mass gain, but 85% experienced mass loss, or a reduced rate of mass gain, before death. Of the 169 nestlings that apparently died from food shortage, 8% were found emaciated but otherwise intact, 18% were partially eaten, and 73% were completely consumed by their siblings or parents.

We had difficulty consistently recapturing individuals from some broods; therefore sample sizes used in analyses requiring growth measurements are lower than those in other analyses. Mean fledgling mass per brood was unaffected by year or supplemental feeding, whether two experimental groups over 3 years were examined or three experimental groups over 2 years (Tables 2 and 3). In all 3 years, owlets were structurally smaller (i.e., lower PC1 values) in unfed broods compared with broods receiving extra food from hatching until fledging (Tables 2 and 3). Likewise, when all three treatments in 1993 and 1996 were analyzed, supplemental feeding had a significant influence on fledgling structural size (Tables 2 and 3).

**Table 2:** Mean (±SE) burrowing owl fledgling mass and structural size (PC1; see text for description) per brood, in relation to year (1992, 1993, and 1996) and feeding treatment in Saskatchewan, Canada

Treatment	Mass (g)	Size (PC1)	# Broods
1992			
Unfed controls	125.7 ± 6.0	−0.92 ± 0.56	4
Fed (hatching to fledging)	138.5 ± 3.0	0.37 ± 0.18	5
1993			
Unfed controls	137.5 ± 5.7	−0.60 ± 0.60	9
Fed (hatching to fledging)	132.2 ± 3.4	−0.27 ± 0.25	10
Fed (laying to fledging)	135.2 ± 1.9	0.02 ± 0.16	14
1996			
Unfed controls	126.5 ± 3.6	−0.70 ± 0.44	6
Fed (hatching to fledging)	137.4 ± 7.9	0.43 ± 0.34	5
Fed (laying to fledging)	133.0 ± 1.6	0.21 ± 0.21	15

Fledgling measures could only be determined for birds from artificial burrows. “# Broods” was the sample size used for statistical comparisons.

**Table 3:** Two-way ANOVA tables for the effects of various supplemental feeding treatments and study year on burrowing owl average brood fledgling mass (g) and structural size

	Mass (g)			Size (PC1)				
*F*	*P*	*F*	*P*
Treatment				
Control	0.64	0.43	4.48	0.04
Fed from hatching to fledging				
Year				
1992	0.19	0.83	0.26	0.77
1993				
1996				
Treatment × Year	2.01	0.14^1^	0.70	0.50
Treatment				
Control	0.07	0.93	2.76	0.07^2^
Fed from hatch to fledging				
Fed from egg laying until fledging				
Year				
1993	0.85	0.36	0.82	0.37
1996				
Treatment × Year	1.92	0.15^3^	0.57	0.57

Because sample sizes were small, we lowered the probability of Type II errors by accepting *P*-values as significant when *P *< 0.10. Interaction terms were initially included, but were all subsequently excluded because they were nonsignificant. Values presented for Treatment and Year were calculated after removal of interaction terms.

1 POWER = 0.53.

2 Tukey tests: Unfed versus Fed (hatching to fledging), *P* = 0.23; Unfed versus Fed (laying to fledging), *P* = 0.05; Fed (hatching to fledging) versus Fed (laying to fledging), *P* = 0.88.

3 POWER = 0.52.

#### Return rates

Average yearly return rate of nestlings was 3%, but return rate was highest in 1998 (8%). Because 1997 was an unusual year in terms of food abundance, we removed return data from 1998 for comparison of return rates of individuals from different treatment groups. Return rate of individuals from fed nests was 2.6% (14 of 526 banded nestlings), whereas return rates of individuals from unfed nests was 1.7% (2 of 113 banded nestlings).

## Discussion

### Timing of food limitation

Our feeding experiments conducted in 1993 and 1996 showed that food limitation was more influential during the nestling stage alone than during earlier stages in the breeding season (i.e., laying and incubation). Observational studies have shown that partial brood loss, through starvation, occurs in a wide variety of birds (reviewed in O'Connor 43; Howe 25). Investigations involving a wide variety of taxa have demonstrated that partial brood loss is reduced by food supplementation during the breeding season (Hogstedt 23; Arcese and Smith 1; Dhindsa and Boag 12; Soler and Soler 58). However, because such feeding experiments extended from before egg laying through fledging, it is difficult to determine the exact period during the nesting cycle when food limited reproductive output. First, extra food prior to clutch initiation typically causes early laying (reviewed in Arcese and Smith 1) and nestling survival often decreases with later CIDs (Perrins 44; Daan et al. 10; Siikamaki 56); therefore, the effects of early laying and food supplementation become confounded. Secondly, when supplemental feeding is conducted throughout the breeding season, it remains unclear if observed increases in fledging success result from alleviation of food limitation during the nestling period or alleviation during earlier stages (Nilsson 41). For instance, supplemental feeding during prelaying and laying can increase egg size (Hogstedt 23; Hill 21; Wiebe and Bortolotti 66), which can in turn increase hatchling size and nestling survival (Martin 36; Perrins 45). Benefits of supplementation can also carry over from one stage to the next if adult condition is affected (Hochachka and Boag 22) or if extra food is stored in caches (Korpimaki 28). Hence, the strongest test of food limitation during the nestling stage is supplementation during that stage alone while controlling for CID and hatching dates.

Given that nestling survival was so high in both food-supplemented groups, perhaps it is not surprising that the proportion of hatchlings to reach fledging age did not differ between those two treatments. However, burrowing owl pairs supplemented with food from the start of laying through fledging also produced the same number of fledglings as pairs supplemented only from hatching to fledging. If pairs had been proximately limited by food prior to the nestling period, extra food during laying or incubation should have allowed pairs to lay more eggs (e.g., Clifford and Anderson 8), or perhaps hatch a higher proportion of their eggs, resulting in more hatchlings (e.g., Korpimaki 28), yet pairs supplemented from the start of laying produced the same number of hatchlings as pairs that received no supplemental food before hatch. This lack of influence of extra food early in the nesting period, measured in terms of number of hatchlings or ultimately number of fledglings, is supported by earlier observations in this same burrowing owl population, showing that egg volume, clutch size, hatching success, and degree of hatching asynchrony were all unaffected by supplemental feeding during laying and incubation (Wellicome 63, 64).

Results from our feeding experiments, and as originally suggested by Lack (29, 30), demonstrated that the ability of parents to meet the energy requirements of nestlings often limits the number of offspring that parents raise. Our experimental design should be implemented using other study species, in other geographic locations and habitats, to determine how generalizable and frequent these patterns of food limitation are for this and other altricial species. If repeated at set intervals over a long time period, this experimental design also could identify any shifts in the timing of food limitation in wild bird populations.

Brood-size manipulation experiments have shown that, in some species, individuals can raise a larger family than they typically hatch under normal conditions (VanderWerf 61). This could result in species or populations for which clutch size or hatching success is most often proximately limited by food intake (e.g., Clifford and Anderson 8). In such species, one would predict that clutch size or hatching success would increase with supplemental feeding during the relevant stage(s); which is indeed the case for some species (Harrison et al. 20), in contrast to results from food supplementation with burrowing owls. It is possible that burrowing owl parents could choose to direct energy from extra food toward increasing their own condition, thereby increasing their own future reproductive potential. However, this seems an unlikely strategy for burrowing owls, given that these owls only breed in 1 or 2 years.

### Quality and quantity of fledglings

For burrowing owls, food consistently limited fledgling structural size. Also, in 1992 and 1996, fledgling mass tended to be higher in supplemented compared with control broods. It remains unclear whether fledgling mass or structural size provided the best index of burrowing owl offspring quality; however, it appears that increased size and mass of fledglings from fed broods did not confer a significant postfledgling survival advantage over individuals from control broods (Todd et al. 60). Overall, we suspect that supplemented pairs likely produced more recruits than control pairs because supplemented pairs fledged many more nestlings than did control pairs, survival of juvenile owls between fledging and migration did not differ between those that received supplemental food during the nestling period and those that did not (Todd et al. 60), and observed return rates were at least the same (or slightly higher) for birds from fed nests compared with those from control nests.

### Reproductive strategy

Many birds show changes in clutch size that correspond to changes in food levels during prelaying and laying periods (reviewed in Martin 36). This pattern of high clutch-size variation, coupled with a low rate of nestling mortality, has been recorded for several owls (e.g., *Strix uralensis*, Lundberg 32; Pietiainen et al. 46; *Bubo virginianus*, Houston et al. 24), indicating that either these species are adjusting their clutch sizes appropriately for predictable posthatch food limitation or their clutches are proximately limited by food. In contrast, burrowing owls are free from food limitation prior to hatching (Wellicome 64), and produce many more hatchlings than they are able to rear under normal food conditions. But why would parents consistently produce extra hatchlings, when they could presumably conserve energy by avoiding such overproduction? We suspect that the function of surplus burrowing owl hatchlings may be to provide parents with extra reproductive value when food availability proves unexpectedly high during the nestling period, as it did in 1997. When food availability is average or below average, culling of an appropriate number of marginal offspring seems to be an appropriate strategy (i.e., brood reduction; Ricklefs 52). An inherent characteristic of this strategy is marked annual variation in fledging success in concert with changes in food availability. The fact that partial brood loss for burrowing owls varied substantially among years, and was virtually eliminated when food was supplemented during the nestling period, and also when small mammal populations peaked in 1997, lends support to the brood reduction hypothesis.

### Management

Gervais et al. (18) demonstrated that when prey populations peaked, burrowing owl population growth was driven by increased fecundity, supporting our general results and observations. Whether long periods without prey irruptions are detrimental to owls in Canada remains to be determined, but it has been suggested that both the frequency and amplitude of prey irruptions on the Canadian prairies have decreased (Poulin et al. 47; the most recent prey irruption since 1997 occurred in 2011, R. G. Poulin, unpubl. data). Long-term management of habitat around breeding burrowing owls favoring predictable prey availability and accessibility during the nestling stage is critical for the conservation of this species (as suggested by Thorup et al. 59 for Little Owl *Athene noctua* Scopoli conservation in Denmark). Much of the North American prairie landscape is composed of homogeneous patches of cropland or grasslands that are planted with exotic grass and forb species that are typically taller and denser compared with grazed pastures during the critical burrowing owl nestling period (7- to 20-day-old chicks; Marsh 34). It is likely that the tall height and high density of introduced grasses and forbs in most hayfields and cropland late in the nestling period could prevent efficient owl foraging (Marsh 34). In addition, intensive cattle grazing negatively impacts some small mammal communities (Bueno et al. 7), which could, in turn, lower food intake for burrowing owls. Management strategies should include maintenance and creation of some degree of vegetation heterogeneity on the landscape surrounding owl nests (e.g., grazing of pastures, mowing of hayfields, or harvesting patterns of cropland and hay fields promoting structural heterogeneity). Structural heterogeneity would provide small mammals with suitable overhead cover, but also some open areas providing access to small mammals for foraging owls (Marsh 34). In addition to long-term management, appropriately timed grazing, harvesting, or mowing to remove overhead vegetation cover in these anthropogenic habitat types, thereby allowing owls access to previously inaccessible prey items, could also be beneficial.

We have provided evidence that supplemental feeding can significantly increase reproductive output in burrowing owls. But, we suggest that supplemental feeding of this population for conservation purposes could only be considered a stop-gap measure and could not be implemented at sufficient a scale to reverse population declines (see Wellicome et al. 65). Rather, supplemental feeding could be a useful strategy when attempting to reestablish owl populations (e.g., Mitchell et al. 38). As a cost-saving measure in these types of reestablishment situations, additional food need only be supplied during the nestling stage.

Dunn et al. (13) stated that supplemental feeding studies have rarely been able to differentiate between the effects of food during laying or posthatch periods on timing of reproduction and reproductive output. Understanding when food limits avian reproductive output is essential for understanding how future (or past) changes in seasonal food availability due to anthropogenic habitat or climate change could influence population growth and phenology (Dunn et al. 13). From an applied perspective, where the capability exists to manage habitat at various times during the breeding season, our results and experimental design provide a unique framework for determining the most efficient and cost-effective period to provide predictable and available food for breeding birds.
